# Supplementary material for: β-Cyclocitral from Lavandula angustifolia Mill. Exerts Anti-Aging Effects on Yeasts and Mammalian Cells via Telomere Protection, Antioxidative Stress, and Autophagy Activation
Source: Antioxidants (Basel). 2024 Jun 12;13(6):715. doi: 10.3390/antiox13060715 (PMC11200722; doi:10.3390/antiox13060715)
Supplement: Supplementary file 1 [file antioxidants-13-00715-s001.zip › antioxidants-3051935-supplementary.pdf]

Supplementary Table S1 Common media and reagent configurations

Media and Reagent	Material Prescription
D-(+)-galactose liquid medium	3% D-(+)-galactose, 2% hipolypeptone and 1% yeast extract
D-(+)-galactose agar medium	3% D-(+)-galactose, 2% hipolypeptone, 2% agar and 1% yeast extract
YPD medium	3% D-(+)-glucose, 2% hipolypeptone and 1% yeast extract
YPD agar medium	3% D-(+)-glucose, 2% hipolypeptone, 2% agar and 1% yeast extract
synthetic defined (SD) medium	0.17% yeast nitrogen base without amino acids and ammonium sulphate, 0.5% ammonium sulphate and 2% D-(+)-glucose
phosphate buffer (PBS)	8 g NaCl, 2.896 g $Na_2HPO_4 \cdot 12H_2O$, 0.02 g KH_2PO_4 and 1 L H_2O
30% glycerol solution	300 μL glycerol and 700 μL PBS
CM solution	DMEM sodium containing 1% penicillin and 1% streptomycin
EM solution	DMEM sodium containing 1% penicillin, 1% streptomycin, 10% horse serum (HS) and 5% fetal bovine serum (FBS)
10% electrophoretic separation gel	4 mL H_2O, 3.3 mL polyacrylamide (30%), 2.5 mL Tris-HCl (1.5 mol/L), 0.1 mL SDS (10%), 0.1 mL AP (10%) and 0.005 mL TEMED
Electrophoretic concentrate	2.7 mL H_2O, 0.67 mL polyacrylamide (30%), 2.5 mL Tris-HCl (1.5 mol/L), 0.1 mL SDS (10%), 0.1 mL AP (10%) and 0.005 mL TEMED

Supplementary Table S2 Yeast strains used in the present study

Yeast strains	Genotype	Source
K6001	*MATa, ade2-1, trpl-1, canl-100, leu2-3, 112, his3-11,15, GAL, psi+, ho::*HO*::CDC6*(at HO), *cdc6:hisG, ura3::URA3 GAL-ubiR-CDC6* (at *URA3*)	Gifted by Professor Michael Breitenbach
Δsod1, *Δsod2*, *Δcat*, *Δgpx*, *Δatg2*, *Δatg32* yeast mutants with K6001 background	Replace the *SOD1, SOD2, CAT, GPx, ATG2* and *ATG32* gene in K6001 with kanamycin gene, respectively	Constructed by Professor Akira Matsuura
BY4741	*MATα, his3Δl, leu2Δ0, met15Δ0, ura3Δ0*	Gifted by Professor Akira Matsuura
YOM36	Prototrophic derivative of BY4742 (*MATα, his3Δ1, leu2Δ0, lys2Δ0, ura3Δ0*)	Gifted by Professor Akira Matsuura
YOM38 containing plasmid pR316-*GFP-ATG8*	Prototrophic derivative of BY4742 (*MATα, his3Δ1, leu2Δ0, lys2ΔO*) containing plasmid pRS316-*GFP-ATG8*	Constructed by Professor Akira Matsuura
S288C	*MATα, SUC2, gal2, mal2, mel, flo1, flo8-1, hap1, ho, bio1, bio6*	Purchased by Bio-sci Plasmid Strain Resources Inc.

Supplementary Table S3 Relative telomere length quantification PCR procedure

Step	Temperature	Time	Number of cycles
Initial denaturation	95 °C	10 min	1
Denaturaiton	95 °C	20 sec	
Annealing	52 °C	20 sec	32
Extension	72 °C	45 sec	
Data acquisition	Plate read		
Optional	Melting curve analysis		1
Hold	20 °C	Indefinite	1

Supplementary Table S4 Primary and secondary antibodies used in the experiments

Type	Cell	Name	Source
Primary antibody	YOM38 yeast	anti-GFP (Green Fluorescent Protein) Rabbit pAb (#598)	Medical & Biological Laboratories, Nagoya, Japan
	3T3 cells	TRF2 (D1Y5D) Rabbit mAb (#13136S)	Cell Signaling Technology, MA, USA
	3T3 cells	TERF2IP (D9H4) Rabbit mAb (#5433S)	Cell Signaling Technology, MA, USA
	YOM38 yeast and 3T3 cells	anti β-Actin Mouse Monoclonal Antibody (#CW0096)	CoWin Biotech, Beijing, China
secondary antibodies	YOM38 yeast and 3T3 cells	horseradish peroxidase-linked goat anti-rabbit IgGs (#CW0103)	CoWin Biotech, Beijing, China
	YOM38 yeast and 3T3 cells	horseradish peroxidase-linked goat anti-mouse LgGs (#CW0102)	CoWin Biotech, Beijing, China

S2. Materials and Methods

S2.1. The methods of lifespan assay

The substances and reagents needed for lifespan assay were acquired from the listed vendors: D-(+)-galactose (Sangon Biotech, Shanghai, China); D-(+)-glucose (Sigma-Aldrich Co., St. Louis, MO, USA); hipolypeptone (Nihon Pharmaceutical Co., Ltd., Tokyo, Japan); yeast extract (Oxoid Ltd., Basingstoke, Hants, UK); agar (Sigma-Aldrich Co., St. Louis, MO, USA); yeast nitrogen base without amino acids and ammonium sulphate (BIDI Medical Device Shanghai Co., Ltd., Shanghai, China); ammonium sulphate (Xilong Chemical Co., Ltd., Guangdong, China).

In the replicative lifespan assay, K6001 yeast cells in the logarithmic growth phase underwent triple washing with PBS, followed by the spreading of approximately 4000 yeast cells onto yeast peptone dextrose (YPD) agar plates containing RES or CYC at concentrations of 0, 0.1, 1 and 10 μM. Incubation of these agar plates occurred for 48 hours at 28 °C. Subsequently, forty microcolonies were chosen at random from the agar plates for examination under an Olympus upright microscope (Olympus Corporation, Tokyo, Japan), with the count of daughter cells produced by a single mother cell recorded. The replicative lifespan assay conducted on Δ*sod1*, Δ*sod2*, Δ*cat*, Δ*gpx*, Δ*atg2* and Δ*atg32* yeast mutants with a K6001 background exhibited similarities to those of the K6001 yeast strain.

In the chronological lifespan assay, YOM36 yeasts were cultured in YPD medium overnight. Yeast with an initial OD_{600} value of 0.01 was then transferred to synthetic defined (SD) medium and exposed to either 1 μM RAP or varying concentrations (0, 0.1, 1 or 10 μM) of CYC (considered day 0). After 72 hours of incubation, approximately 200 yeast cells from each group were spread onto YPD agar plates and left to incubate for 48 hours. The colony-forming units (CFUs) on each plate were tallied. This process was repeated every two days until the survival rate (calculated as CFUs divided by CFUs on day 3, multiplied by 100%) fell below 5%.

S2.2. Measurements of Yeast under Oxidative Stress

The yeast's resilience to oxidative stress was evaluated following our previous methodology [32]. BY4741 yeast cultures with an OD_{600} value of 0.1 were grown in YPD and subjected to treatment with either 10 μM RES or varying concentrations (0, 0.1, 1 and 10 μM) of CYC. Following incubation at 28 °C with shaking for 24 hours, 5 μL of each yeast culture with an OD_{600} value of 1.5 was dispensed onto YPD agar plates supplemented with 10 mM H_2O_2. After 72 hours of incubation, yeast growth in each group was documented photographically. For quantitative analysis, BY4741 yeast strains were exposed to RES at a concentration of 10 μM or CYC at concentrations of 0, 0.1, 1 and 10 μM. After 24 hours of incubation, nearly 200 yeast cells were plated onto YPD agar plates with or without 6.2 mM H_2O_2. The survival rate for each group was determined after 48 hours of cultivation, calculated as the number of colonies growing on medium with H_2O_2 divided by the number of colonies growing on medium without H_2O_2, multiplied by 100%.

S2.3. Measurements of ROS and MDA Levels

In the assessment of ROS levels, BY4741 yeast cultures, starting with an initial OD_{600} value of 0.1, were exposed to either 10 μM RES or varying concentrations (0, 0.1, 1 and 10 μM) of CYC for 24 hours. Subsequently, the yeast cells were washed thrice with PBS. A final concentration of 10 μM of the fluorescent probe 2′,7′-dichlorodihydrofluorescein diacetate (DCFH-DA) was added, and the mixture was incubated in the dark with shaking (180 rpm, 28 °C) for 1 hour. Afterward, the yeast cells were collected via centrifugation and washed thrice with PBS to eliminate excess DCFH-DA. The fluorescence intensity of 2′,7′-dichlorodihydrofluorescein (DCF) from approximately 1×10^7 yeast cells in each group was measured at an excitation wavelength of 488 nm and an emission wavelength of 525 nm using a Varioskan Flash spectral scanning multimode reader (Thermo Fisher Scientific, Waltham, MA, USA).

In the assessment of MDA levels, briefly, yeast cells were cultured similarly to those for ROS measurement. Following three washes with PBS, 500 μL of PBS and grinding beads were

introduced, and the mixture was ground for 1 minute at 70 *Hz* utilizing an automated sample rapid grinder (Shanghai Jingxin Inc., Shanghai, China). Subsequently, the disrupted cells underwent centrifugation at 12,000 rpm for 10 minutes at 4 °C, yielding supernatants collected as protein samples. Protein concentrations were determined using a BCA kit (CoWin Biotech, Beijing, China). 100 μL of ethanol, 10 nmol/mL standard or test samples were added to 1.5 mL Eppendorf tubes respectively. The tubes were vortexed well after adding 100 μL reagent I. Subsequently, 375 μL reagent II and 125 μL reagent III were added into the tubes in turn. Finally, these tubes were sealed and heated in a water bath for 80 min at 95 °C. Then, 200 μL of supernatant was taken into each well of a 96-well plate after centrifugation (3500-4000 ×g/min, 10 min), and the absorbance at 532 nm was measured using a BioTek microplate reader (BioTek, Winooski, VT, USA). MDA content in yeast (nmol/mg protein) = (determination group OD value / [standard group OD value−blank group OD value]) × standard concentration (10 nmol/mL) ÷ protein concentration of sample (mg protein/mL).

S2.4. Determination of SOD, GPx and CAT Antioxidant Enzyme Activities

To conduct the assays for SOD, GPx and CAT antioxidant enzyme activities, we first cultured the BY4741 yeast strain in YPD for 24 hours at 180 rpm and 28 °C. Following this, the yeast cells, initially at an OD_{600} value of 0.1, were treated with either 10 μM RES or varying concentrations (0, 0.1, 1 and 10 μM) of CYC. They were then cultured at 28 °C with agitation for another 24 hours. Subsequently, cells from each treatment group were harvested separately. To each sample, 500 μL of PBS and grinding beads were added, and sonication was performed for 1 minute on ice. Protein extraction was achieved by centrifugation at 12,000 rpm for 10 minutes at 4 °C. After determining the protein concentration, each sample was diluted to 1.25 μg/μL for the assessment of antioxidant enzyme activities. Finally, the enzyme activities in each group of yeast cells were determined following the instructions provided in the SOD (Nanjing Jiancheng Bioengineering Institute, Nanjing, China), GPx and CAT (Beyotime Biotech, Shanghai, China) Antioxidant Enzyme Activity Assay Kits.

For SOD enzyme activity assay, 25 μg protein in each group was first mixed with reagent VII and vortexed for 1 min to inactivate the Mn-SOD enzyme activity in the samples. The supernatant was obtained for detecting the CuZn-SOD enzyme activity after centrifugation (3500× rpm, 15 min). The reagent I, blank control, samples, and the samples treated by reagent VII were added to the 96-well plate according to the dosage in the manufacturer instructions. Then, reagents II, III, and IV were added into each well. Then, the plate was incubated at 37 °C for 40 min after mixing well. Finally, the A550 absorbance value of samples was measured after reacting with 120 μL of chromogenic working fluid at room temperature for 10 min under dark. Activity of SOD enzyme = ([control group OD value - determination group OD value] / control group OD value) / 50% × (total volume of reaction solution / sample volume)/ protein concentration of sample.

During CAT enzyme activity assay, gradient concentrations of hydrogen peroxide solution was first prepared. Afterward, chromogenic working fluid was added to the 96-well plate to mix with hydrogen peroxide solution and reacted at 25 °C for 15 min. The standard curve of hydrogen peroxide concentration was determined after measuring the absorption value at 520 nm. Simultaneously, catalase buffer and 250 mM of hydrogen peroxide were added to each well with 5 μL protein samples (1.25 μg/μL). After reacting at 25 °C for 1–5 min, 450 μL of enzyme reaction termination solution was added to terminate the reaction. Then, 10 μL mixture was taken to react with chromogenic working fluid at 25 °C for 15 min, and the absorption value at 520 nm was measured. Sample catalase activity = [consumption of micromole of hydrogen peroxide] × [dilution ratio] / ([reaction minutes] × [sample volume] × [protein concentration]), and [consumed micromole of hydrogen peroxide] = [micromole of residual hydrogen peroxide in blank control] − [micromole of residual hydrogen peroxide of sample].

For GPx enzyme activity assay, 12.5 μg protein of each sample was taken. The general process is that the GPx detection buffer, samples, GPx detection working solution, and peroxide reagent were added in a 96-well plate in turn. The absorbance value of A_{340} was measured every 3 min and for six times after mixing well. The activity of GPx in the detection system = [(ΔA_{340} (sample) − ΔA_{340} (blank))/min] / (0.00622 $\mu M^{-1} cm^{-1}$ × 0.276 cm). Total GPx activity in the sample = GPx activity in the detection system × dilution ratio / sample protein concentration.

Supplementary Figures

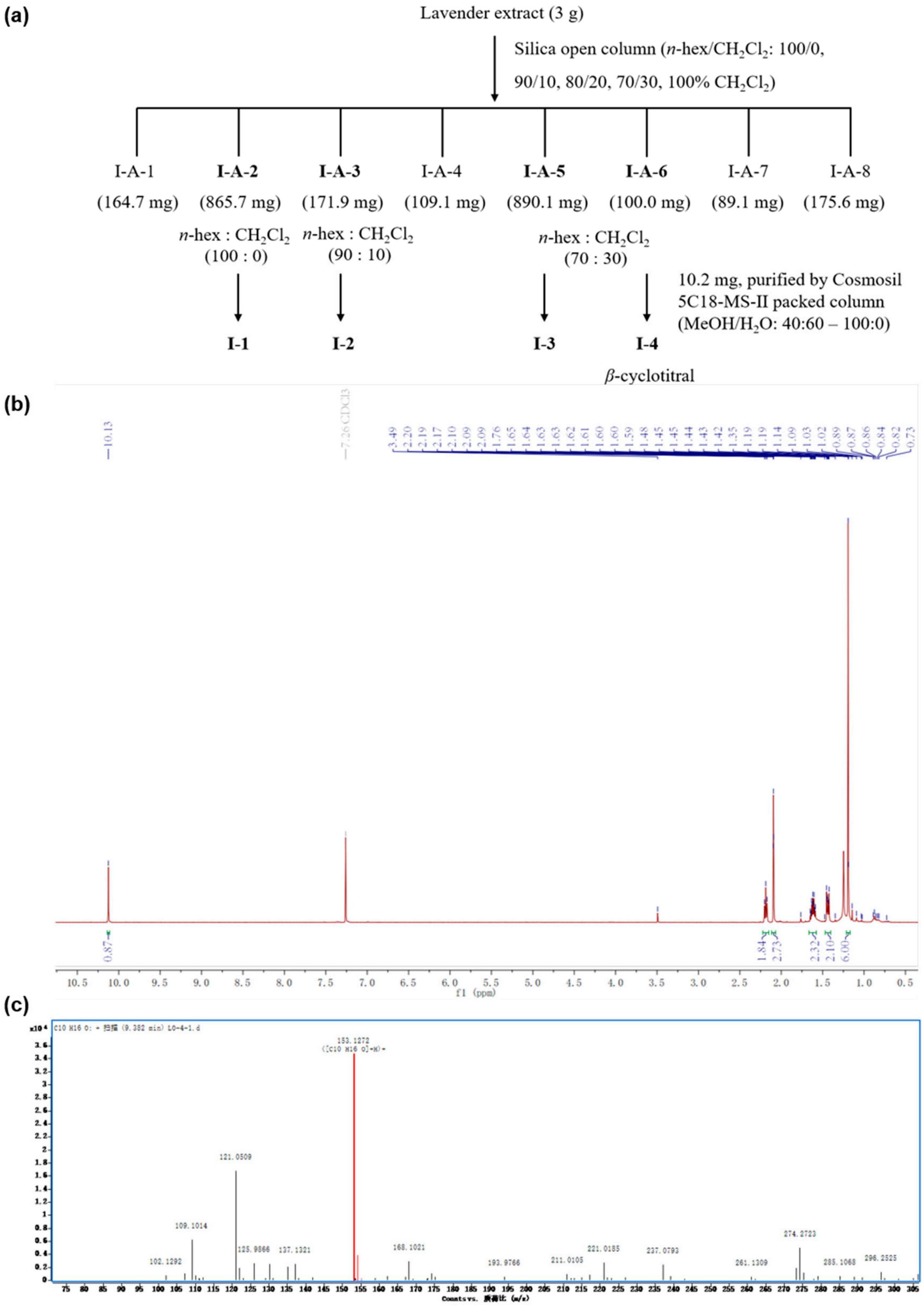

Supplementary Figure S1. The isolation procedure and physicochemical data of CYC. (a) The details of isolation procedure of CYC. Silica gel (200-300 mesh, Yantai Research Institute of Chemical Industry, Yantai, China) and Cosmosil 5C18-MS-II packed column (*Φ*10/250 mm, Nacalai Tesque, Kyoto, Japan) were utilized for the isolation and purification from lavender essential oil. **(b)** The ^{1}H NMR spectra and **(c)** HR ESI-TOF-MS data of CYC.

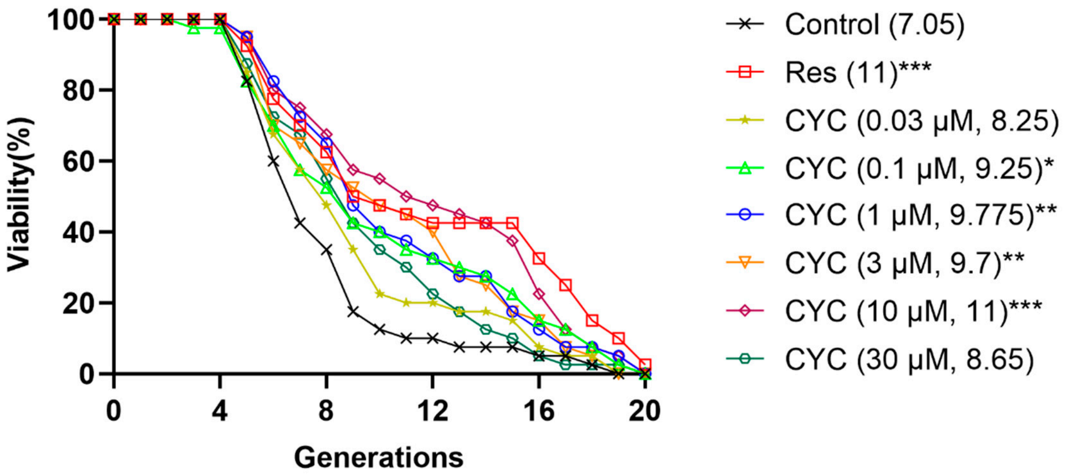

Supplementary Figure S2. Effect of CYC on the replicative lifespan of K6001 yeasts. The average lifetime of each group was as follows: control (7.05 ± 0.53), RES at a dose of 10 μM (11.00 ± 0.87), CYC at a dose of 0.03 μM (8.23 ± 0.65), CYC at a dose of 0.1 μM (9.25 ± 0.79), CYC at a dose of 1 μM (9.76 ± 0.68), CYC at a dose of 3 μM (9.70 ± 0.71), CYC at a dose of 10 μM (11.00 ± 0.77), CYC at a dose of 30 μM (8.65 ± 0.60). *, ** and *** represent significant differences compared to the control group at $p < 0.05$, $p < 0.01$ and $p < 0.001$, respectively.

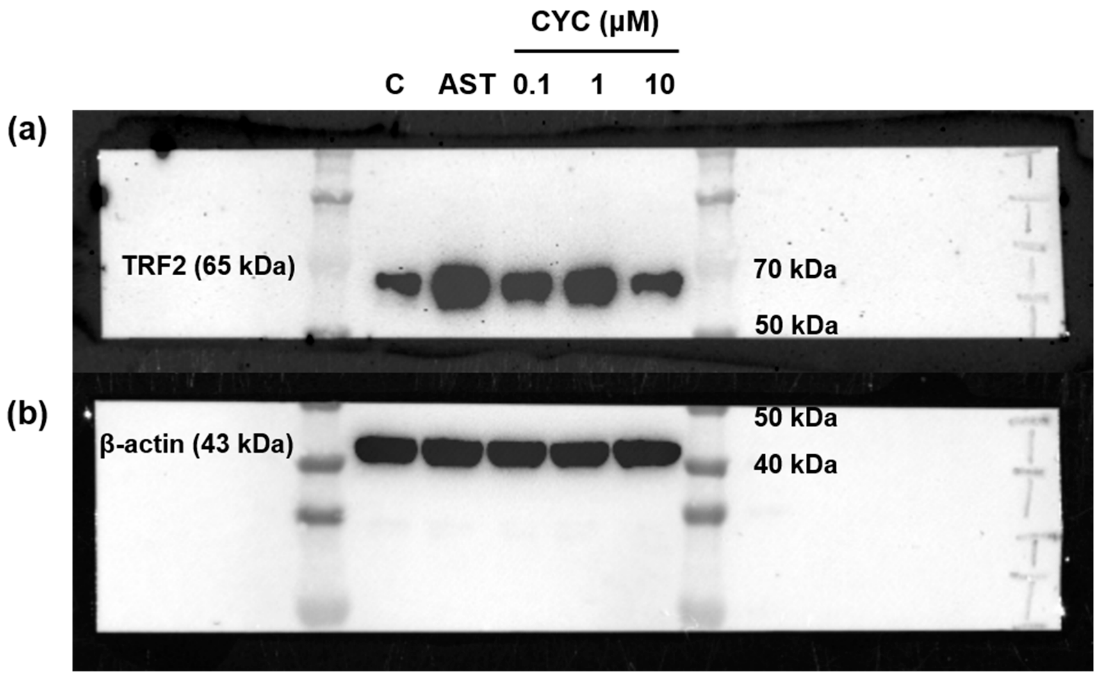

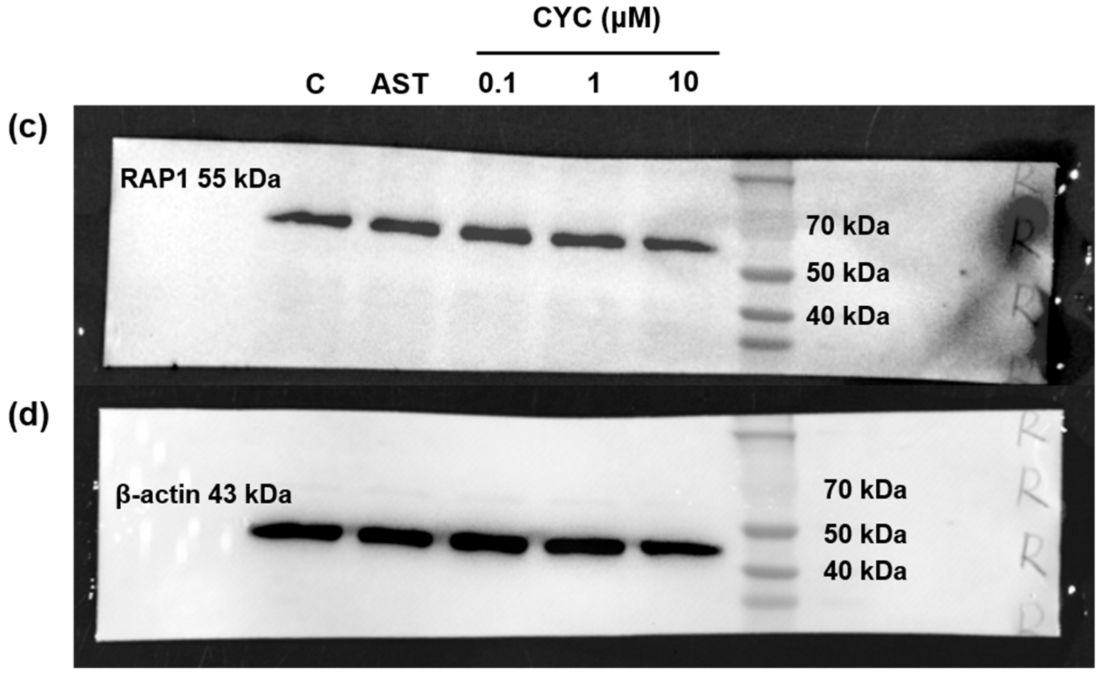

Supplementary Figure S3. The full unedited gel for western blot analysis of TRF2, RAP1 and *β*-actin in yeast in Figures 2c, e. **(a)** The TRF2 and *β*-actin are obtained from cutting one membrane by loading the same protein **(b)** The RAP1 and *β*-actin are obtained from one membrane by using stripping buffer**.** Protein bands are obtained by exposure via Bio-Rad chemiluminescence imager (Bio-Rad Laboratories, Hercules, California, USA).

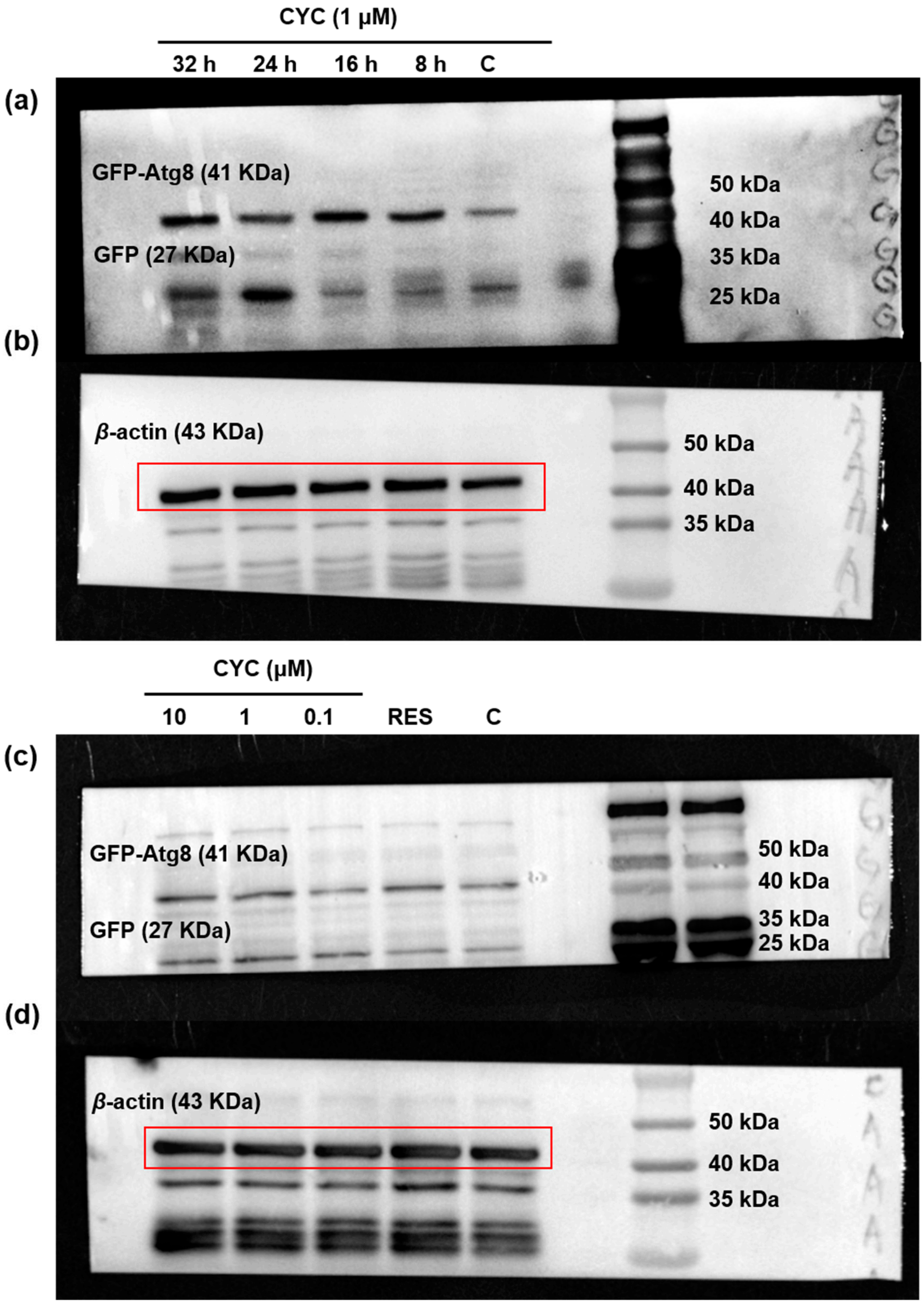

Supplementary Figure S4. The full unedited gel for western blot analysis of GFP-Atg8, free GFP, and β-actin in yeast in Figures 6e, g. (a) (b) The GFP-Atg8, free GFP and β-actin are obtained from two membranes by loading same amount of protein. Protein bands are obtained by exposure via Bio-Rad chemiluminescence imager (Bio-Rad Laboratories, Hercules, California, USA).
